# Vascular Pathology and Blood-Brain Barrier Disruption in Cognitive and Psychiatric Complications of Type 2 Diabetes Mellitus

**DOI:** 10.1155/2011/609202

**Published:** 2011-02-17

**Authors:** Yonatan Serlin, Jaime Levy, Hadar Shalev

**Affiliations:** ^1^Department of Physiology and Neurobiology, Faculty of Health Sciences, Ben-Gurion University of the Negev, Beer-Sheva 84105, Israel; ^2^Department of Ophthalmology, Soroka University Medical Center, Ben-Gurion University of the Negev, Beer Sheva 84105, Israel; ^3^Department of Psychiatry, Soroka University Medical Center, Ben-Gurion University of the Negev, Beer-Sheva 84105, Israel

## Abstract

Vascular pathology is recognized as a principle insult in type 2 diabetes mellitus (T2DM). Co-morbidities such as structural brain abnormalities, cognitive, learning and memory deficits are also prevailing in T2DM patients. We previously suggested that microvascular pathologies involving blood-brain barrier (BBB) breakdown results in leakage of serum-derived components into the brain parenchyma, leading to neuronal dysfunction manifested as psychiatric illnesses. The current postulate focuses on the molecular mechanisms controlling BBB permeability in T2DM, as key contributors to the pathogenesis of mental disorders in patients. Revealing the mechanisms underlying BBB dysfunction and inflammatory response in T2DM and their role in metabolic disturbances, abnormal neurovascular coupling and neuronal plasticity, would contribute to the understanding of the mechanisms underlying psychopathologies in diabetic patients. Establishing this link would offer new targets for future therapeutic interventions.

## 1. Introduction: The Vascular Hypothesis

Macro- and microvascular complications involving endothelial dysfunction are central to the pathogenesis and clinical manifestations of type 2 diabetes mellitus (T2DM) [1]. Structural brain abnormalities [2–7] and cognitive, learning and memory deficits were demonstrated in T2DM patients [8–10]. We recently published a hypothesis paper suggesting that a primary vascular pathology involving inflammatory cascade and Blood-Brain Barrier (BBB) breakdown, will result in the leakage of serum-derived vascular components into the brain tissue and may cause brain dysfunction which, under some conditions (extent, duration, and/or location), will result in disturbed thinking processes, mood, and behavior, such as those characterizing psychiatric illnesses [11]. The current postulate focuses on inflammation and molecular mechanisms controlling BBB permeability in T2DM as key contributors to the pathogenesis of mental disorders in diabetic patients and suggests novel targets for the prevention and treatment of cognitive and psychiatric complications.

## 2. Type 2 Diabetes Mellitus and Vascular Pathology

T2DM is a multifactorial metabolic disorder. The underlying etiology, pathophysiology and complications of diabetes are still being elucidated (for review see [12]). T2DM is characterized by chronic abnormal high blood glucose levels (hyperglycemia), insulin resistance, and a relative insulin secretion defect [13]. Induction of insulin resistance is linked to obesity and activation of neuroendocrine and inflammatory responses [14–16]. Approximately 200 million people worldwide have diabetes and it is estimated that without proper measures to slow the epidemic advance of the disease, by 2025 the number of patients will increase to 333 million [17]. T2DM is recognized as an independent risk factor for cardiovascular disease (CVD), presenting increased risk of morbidity and mortality from coronary heart disease, congestive heart failure, and stroke [18]. Accumulating clinical data disclose the central role of vascular lesions and inflammation in the pathogenesis of T2DM and associated complications [19]. Diabetic macrovascular complications involve vessel obstructions, such as coronary artery diseases, atherosclerosis, and peripheral vascular diseases. Microvascular pathologies include retinopathy, nephropathy, and neuropathy [20]. Direct damage to small blood vessels, particularly by hyperglycemia, is manifested by endothelial dysfunction, diminished perfusion, abnormal endothelial cell (EC) proliferation and increased vessels permeability [21]. T2DM patients exhibit similar microvascular damage within the central nervous system (CNS) which may result in increased incidence of cognitive deterioration, vascular dementia, lacunar infarcts, hemorrhages and Alzheimer's disease [22].

## 3. Structure and Function of the Blood-Brain Barrier and Quantification of Its Disruption

First evidences for a barrier preventing the passage of water-soluble dyes from the circulation to the brain tissue and the spinal cord were presented consecutively by Ehrlich, Goldmann and Lewandowsky in the beginning of the 20th century [23]. At the interfaces between the blood and the neural tissue or its fluid spaces exist three barrier layers: (1) the BBB present in the capillaries throughout the brain, formed by highly specialized EC partitioning between the blood and brain interstitial fluid, (2) the choroid plexus epithelium between blood and ventricular cerebrospinal fluid (CSF), and (3) the arachnoid epithelium between the blood and subarachnoid CSF [24]. The BBB components include the EC with their basement membrane, lining the lumen of brain capillaries. EC adjoined by specific protein tight junctions (e.g., claudins, occludins, ZO-1, ZO-2, ZO-3 and cingulin) and display specific transport mechanisms and pinocytic vesicles (for review see [25]). The endothelium is enclosed by brain pericytes and astroglial foot processes which form a third continuous layer that separates these blood vessels from the brain tissue. Jointly, these components form a barrier that hinders the entry of most molecules into the brain, and enable active transportation of penetrated molecules out of the brain. Common brain imaging methods, such as magnetic resonance imaging (MRI), computerized tomography (CT), and single photon emission CT (SPECT) are employed for qualitative evaluation of BBB disruption in patients. Extravascular accumulation of a peripherally administrated nonpermeable contrast agents, indicate BBB breakdown [11]. Several methods for quantification of BBB permeability using dynamic contrast enhanced imaging were developed, although a routine clinical examination is not yet available [26–28]. In the clinical setting, quantitative evaluation of BBB disruption can be held by CSF analysis for serum proteins (e.g., albumin) or plasma analysis of brain constituents (e.g., S100B-brain-specific astrocytic calcium-binding protein) [29].

## 4. Mechanisms of BBB Breakdown

BBB integrity is altered in diverse pathological conditions. Changes are manifested by disruption of junctional components which result in transbarrier leakage, and BBB activation, which relates to the expression and secretion of immune factors by its cellular components. The underlying molecular changes leading to BBB dysfunction are not completely clear, but may involve amplification of endothelial caveolae leading to transcytosis of plasma proteins [30, 31], decreased expression of junctional adhesion as well as tight junction proteins [32, 33], and increased expression of matrix metalloproteases [34]. Reactive cellular activity in the neurovascular junction has also been observed, including increase in migratory activity of pericytes [35] and the proliferation of blood vessels due to upregulation of vascular endothelial growth factor (VEGF) [36]. BBB opening itself leads to the exposure of the brain tissue to serum-derived (normally nonpermeable) molecules, which serve as signaling mediators for brain repair mechanisms but may also facilitate BBB breakdown. Agents released during inflammation aggravate the penetrability of the brain endothelium. EC bradykinin B2 receptors activation lead to an increase in intracellular Ca2^+^ concentrations [37] and subsequently to activation of endothelial nitric oxide synthase (eNOS) which promotes transient tight junctions opening and increased permeability [38]. Furthermore, bradykinin can activate NF-*κ*B pathway in astrocytes, leading to the release of interleukin-6 (IL-6), which can amplify the effect by acting back on the endothelium [39]. Tumor necrosis factor-*α* (TNF*α*) increases BBB permeability by direct action on the endothelium [40] and indirectly via endothelin-1 production and IL-1*β* release from astrocytes [41]. Mediators released from central and peripheral cellular components and connective tissue following injury, can also affect BBB permeability. For example, histamine and TNF*α* and interferon-*γ* released in inflammatory pain can alter brain endothelial permeability [42]. IL-1*β* release may lead to a decreased concentration or altered localization of the tight junction protein occludin, and thus increases BBB permeability. Metalloproteases causing BBB breakdown are upregulated and released during spreading neuronal depolarization after massive neuronal activation [43].

## 5. Blood-Brain Barrier Breakdown in Diabetes Mellitus

### 5.1. Anatomical Changes

Altered BBB structure in diabetic patients is a matter of debate. Several studies have indicated that the BBB integrity is sustained in DM, while others revealed association between DM and increased BBB permeability. Intravital microscopy examination of BBB integrity in diabetic rats using fluorescent-labeled albumin displayed intact BBB [44]. These findings should be interpreted with caution since intravital microscopy for quantification of ligands extravasations through the BBB is often complicated and not specific [45]. Postmortem examination of prefrontal and temporal cortex of diabetic patients together with immunohistochemical stainings against serum proteins concluded that the BBB is well maintained [46]. In contrast, growing body of evidence propose an opposing notion. Animal models of ischemic injury in diabetic rats demonstrated that hyperglycemia significantly aggravated BBB permeability, edema formation, and neurological manifestations [47, 48]. BBB breakdown after ischemia/reperfusion injury result in extravasation of inflammatory cells and fluid into the brain tissue, and thus suggest that BBB disruption has important role in the pathogenesis of brain damage associated with systemic hyperglycemia. MRI brain imaging following intravenous gadolinium administration identified increased BBB permeability in diabetic patients compared to controls [49]. Antibodies against serum S100B and NSE (CNS proteins) were found to be significantly increased in both type 1 and type 2 diabetic subjects compared to controls, implying that diabetes in humans may be associated with alterations in the integrity of the BBB [50].

### 5.2. Metabolic Changes

Normal metabolic activity of neural tissue relies on constant glucose delivery. Due to the high metabolic demands, glucose transport from the blood across the BBB into the cells of the brain is mediated by rapid facilitated transport. Glucose transporter proteins (GLUT), particularly GLUT1 and GLUT3 ensure glucose supply. GLUT1 protein is highly expressed at the BBB and GLUT3 is primarily found in neurons. GLUT1 expression is controlled by blood glucose levels, to maintain sufficient distribution for optimal neuronal function [51]. In diabetes, imbalance of glucose metabolism, lead to alterations of glucose transport into the brain. Pardrdige et al. (1990) [52] showed a decrease in GLUT1 expression and activity in diabetic rats thus leading to reduced glucose transport in uncontrolled diabetes. Studies focused on chronic hyperglycemia and increased vascular damage showed that abnormal glucose metabolism results in generation of reactive oxygen species (ROS) followed by oxidative stress, mitochondrial dysfunction and inflammatory response [53, 54]. Hyperglycemia is presumed to play a role in the generation of acute phase proteins and inflammatory response [55]. It correlates with data about reduction of the levels of acute-phase serum proteins by treatments that increase insulin sensitivity and lower blood glucose [56].

### 5.3. Inflammatory Mechanism: From Diabetic Retinopathy to Brain Pathology

#### 5.3.1. Inflammatory Mechanisms

Inflammatory mechanisms underlying vascular pathology in DM are possibly common to the vasculature in the periphery and CNS. Formation of advanced glycation end products (AGEs) via glycation of blood proteins is a consequence of hyperglycemia, and it results in decreased kidney function and small vessels pathology. AGEs accumulation may induce vascular inflammation by the interactions between AGEs and AGE-specific receptors (RAGE) [57]. AGEs activation of endothelial RAGE promotes upregulation of endothelial adhesion molecules including vascular cell adhesion molecule 1 (VCAM-1) and activates transcription factor nuclear factor-*κ*B (NF*κ*B). The former increases monocyte adhesiveness and vascular permeability while the latter regulates multiple proinflammatory and proatherosclerotic target genes in endothelial and vascular smooth muscle cells as well as in macrophages [58].

#### 5.3.2. Diabetic Retinopathy

Well-established data about retinal vessels pathology in DM is available. Due to the structural similarities between the BBB and the blood-retinal barrier (BRB) and the fact that disruption of the BRB in diabetes is associated with retinopathy, it is logical to assume that altered BBB function in DM patients may also result in brain pathology. Chronic hyperglycemia, hyperlipidemia, and hypertension contribute to the pathogenesis of Diabetic Retinopathy (DR) [59–61]. Diabetic macular edema (DME) found in 29% of patients who had diabetes for ≥20 years [62] and is caused by increased level of mediators responsible for retinal vascular permeability as IL-6 and VEGF. These factors promote leakage of intravascular fluid from retinal capillaries into retinal spaces [63]. Further damage arises from retinal EC exposure to AGEs leading to abnormal eNOS expression [64] and induction of VEGF expression [65]. Diabetes may also involve altered retinal blood flow as an outcome of the damage to pericytes enclosing the BRB [66, 67] and correlates with microaneurysm formation. Capillary nonperfusion, EC damage, and vessel occlusions contribute to the retinal microcirculation damage [68]. Capillary occlusion by leukostasis, adherence to the vascular endothelium and cellular degeneration lead to retinal ischemia that stimulates pathologic neovascularization mediated by angiogenic factors (e.g., VEGF) which enhance BRB permeability and result in proliferative diabetic retinopathy (PDR) [69, 70]. During the last years, anti-VEGF drugs, such as ranibizumab (Lucentis) and bevacizumab (Avastin) are injected into the vitreous for the treatment of diabetic macular edema.

#### 5.3.3. BRB Examination as a Window for BBB Condition

As previously described, the vascular hypothesis speculates that BBB disintegration may be involved in the pathogenesis of brain diseases. Diabetes-induced microangiopathy of the kidney and retina are well described in the literature. The detailed pathogenesis of microvascular damage within the CNS is less known, since altered functions of cerebral vessels is concealed and less predictable, while in other tissues vascular impairments are detectable and obvious [71]. The analogy between the BBB and the BRB is the platform for conceptualization that retinal vessels examination can provide a tool for estimation of cerebral vessels status. In the clinical setting, investigation and documentation of the BRB integrity are held routinely in T2DM patients. Ophthalmic fluorescein angiography (FA) includes intravenously administration of fluorescein producing angiographic display that is used to visualize retinal blood flow dynamics while recording the integrity of the BRB. Correlation between FA results and BBB permeability measures utilizing dynamic contrast enhanced imaging (e.g., [26, 72]) is thus essential, in order to point out the mutual relation between the two systems. An important feature should be the ability to quantify BRB leakage in T2DM patients, and novel imaging methods can be implemented [73]. Future perspectives should focus on developing novel applicable tool for prediction of BBB breakdown via BRB image analysis. A similar conclusion was published recently, following the results of a prospective study using MRI examination and retinal imaging [74]. It has been shown that retinal microvascular abnormalities are associated with emergence of subclinical brain infarcts and white matter lesions, and proposed that retinal vascular imaging may offer a noninvasive tool to investigate cerebral small-vessel disease.

## 6. Blood-Brain Barrier Breakdown in Psychiatric Diseases

There are evidences linking psychiatric illness with BBB alterations. Quantitative evaluation of BBB disruption utilizes CSF analysis for presence of serum proteins leaked through a permeable barrier, or plasma analysis for molecules found exclusively in the brain (as S100B). Similarly, increase in plasma levels of S100B may reflect increased BBB permeability [29]. CSF/serum albumin ratio was elevated in patients suffering from dementias, in comparison to nondemented individuals [75] and in elderly depressed women compared to women without depression [76]. BBB dysfunction was also shown in schizophrenic patients by measuring increased albumin and IgG CSF levels, with additional correlation between the negative symptomatology to CSF/serum albumin ratio [77, 78]. Bell and Zlokovic (2009) recently reviewed the knowledge about the relation between cerebrovascular dysfunction as BBB disruption and neurovascular uncoupling, to cognitive decline and neurodegenerative changes of Alzheimer's disease [79]. Clinical studies demonstrated increased S100B levels in the serum of patients suffering from acute or chronic schizophrenia [80]. Same serum S100B elevation was observed in patients with major depression, with decrease in serum S100B levels during clinical improvement after antidepressant treatment [81].

## 7. Comorbidity between Diabetes Mellitus and Psychiatric Disorders

Among DM patients there is a significant and consistent association with presence of elevated depressive symptoms and the prevalence of major depression, compared with the general population [82–85]. Recently published data shows that higher A1C levels are associated with lower cognitive function in individuals with diabetes [86]. Accumulating evidence [9, 10] indicates that in diabetic patients, hyperglycemia and diabetes durations contribute to brain atrophy and increases the risk of cognitive impairment. Increased expression of RAGE in Alzheimer's disease brain, indicates its relevancy in the pathogenesis of neuronal dysfunction and death [87]. Postmortem studies of individuals with Alzheimer's disease attributes to this opinion by demonstrating AGEs within the senile plaques [88, 89]. Indeed, studies suggest that T2DM is associated with an increased risk of Alzheimer disease, vascular dementia and risk for development of cognitive impairment in comparison with the general population [90–92]. Anxiety disorders were also found in high prevalence in diabetic population [93, 94].

## 8. Inflammation and Psychopathology

 Inflammatory processes are central to the pathogenesis of T2DM and contribute to BBB dysfunction. Apart from the pathogenic role of the immune responses, accumulating data indicates that immunologic responses also play a role in depression, neurodegeneration, and deficits in cognitive function. Evidence of an inflammatory response in major depression is present over the last two decades [95]. Recent meta-analysis of 24 studies reinforced the notion about cytokine involvement in depression through activation of the inflammatory response [96]. A thorough review by Maes et al. (2009) [97] elaborates the involvement of inflammatory pathways in depression. Increase in proinflammatory cytokines, such as IL-1*β*, IL-6, interferon-*γ* and TNF*α*, with a relative shortage in the anti-inflammatory cytokine IL-10 was documented in depression. Cytokines produced in the periphery and by neurons and glial cells within the CNS are presumed to be involved in the complex autonomic, neuroendocrine, metabolic and behavioral responses to brain injuries as inflammation, ischemia and stroke [98–100]. As mentioned previously, in T2DM, inflammation of adipose tissue contributes to insulin resistance. Activated macrophages in the adipose tissue are the primary cellular source of proinflammatory cytokines as IL-1*β*, TNF-*α* and IL-6. These mediators provide additional links between the participation of immune reaction in T2DM and the brain response. In brain regions lacking intact BBB (i.e., circumventricular organs), cytokines leakage from the blood into the brain parenchyma may lead to activation of macrophages and induction of a proinflammatory cascade. Additionally, without crossing the BBB, cytokines are able to interact with perivascular macrophages (reviewed by [101]). Clinical data from patients with major depression demonstrate increase of inflammatory features among them [102]. Studies pointing out the existence of positive correlations between plasma concentrations of inflammatory mediators and the severity of depressive symptoms are also available [103, 104]. Proinflammatory response induces decreased neurogenesis in depression, which is characterized by decreased brain-derived neurotrophic factor (BDNF), neural cell adhesion molecule (NCAM) and fibroblast growth factor (FGF) [105–107]. Inflammation stimulates release or production of corticotropin releasing hormone (CRF), adrenocorticotropic hormone (ACTH) and cortisol via activation of the hypothalamic-pituitary-adrenal axis (HPA) and cortisol in turn may participate in neural atrophy [108, 109]. Furthermore, inflammatory cytokines as IL-1*β*, IFN*γ*, and TNF*α* cause induction of indoleamine-2,3-dioxygenase (IDO), an enzyme catabolizing tryptophan into neurotoxic metabolites known as TRYCATs. IDO activation is significantly related to inflammatory signs and to the severity of depressive symptoms [110, 111]. Serotonin levels are affected by inflammation since tryptophan is the precursor of 5-HT. IDO metabolize tryptophan in the kynurenine pathway and therefore less tryptophan is available to synthesize 5-HT. Activation of the brain's microglia by Th1 cytokines, either secreted from activated astrocytes or from the periphery, induces IDO and may thus reduce 5-HT levels and result in depression. Astrocytic activation in the brain, facilitated by BBB disruption in inflammatory condition of T2DM may also alter network properties and neuronal excitability by changing glutamate levels and affecting synaptic plasticity. Cytokines may generate, through the kynurenine pathway, the formation of quinolinic acid—an NMDA receptor (NMDAR) agonist. Microglia are the only cells in the CNS that express the complete enzymatic pathway required for the synthesis of quinolinic acid [112]. Hence, inflammatory mediators acting on microglia will increase the levels of quinolinic acid and will activate NMDA receptors. These findings match with new evidence suggesting that heightened glutamate receptor activity in major depression, can underlie inflammation-associated depressive disorders [113]. In addition, quinolinic acid directly causes release of glutamate [114]. Thus, inflammatory mediators can lead to an environment of excess glutamate. Glutamate receptor activation enhances the effect of BBB breakdown by induction of astrocytic transformation. A vicious cycle of cytokine secretion, microglial activation, and further enhancement of glutamate receptors activation is created (see below). Activated microglial cells are also key contributors to the inflammatory response which occur during chronic neurodegeneration in diseases such as Alzheimer's disease, prion disease and Parkinson's disease [115]. These activated microglia release proinflammatory cytokines which affect injured neurons and may exacerbate lesion size and neuronal loss. Postmortem examination of brain tissue from patients suffered from Alzheimer's disease revealed large numbers of activated microglia associated with the amyloid deposits and in regions of the brain where there is neuronal loss [116]. Metabolic syndrome, T2DM, and decline in cognitive function share common inflammatory markers [117]. Elevated levels of insulin may lead to cognitive decline via the effect of hyperinsulinemia on neuronal metabolism and reduced clearance of *β* amyloid, a frequent pathologic feature of obesity, metabolic syndrome, DM, and Alzheimer's disease [118]. A Recent study showed decrease in executive and processing function among metabolic syndrome patients [119]. Moreover, patients with impaired insulin function were found to have lower levels of the neurotrophic protein BDNF. Decreases in hippocampal BDNF levels showed association with stress-induced depressive behaviors and conversely, antidepressant treatment enhanced the expression of BDNF [120]. 

## 9. BBB Breakdown and Psychopathology

Neuropsychiatric disorders such as depression, mood and anxiety disorders, are associated with cerebrovascular impairments [121]. BBB breakdown will result in induction of signaling pathways leading to transformation and activation of the surrounding cells. We mentioned previously how local inflammatory brain responses following BBB changes influence endothelial and glial cells towards elevation of cytokine expression. It is possible to assume that glial cell activation will also participate in the functional changes occurring in the vascular environment and the adjacent neuropil. Indeed, compromised BBB results in a rapid transformation of the resting astrocytes into their active form in ischemic, inflammatory and traumatic brain injuries. Astrocytic endfeet are considered an integral part of the BBB and surround capillaries in the CNS to regulate the vascular tone [122] and tight junction expression [123]. Experimental evidence suggests that upon BBB breakdown, infiltration of albumin, the most abundant serum protein, into the neuropil may account for the astrocytic transformation via the transforming growth factor beta (TGF*β*) signaling pathway. Transformed astrocytes undergoes modification in gene expression that includes the upregulation of GFAP and S100B, downregulation of glutamate transporters, glutamine synthase and the inward rectifying potassium channel (K_IR4.1_), AQP4 and gap junctions' proteins [124, 125]. The subsequent gene expression affects the extracellular environment through increased concentrations of potassium and glutamate causing amplification of neuronal excitability [126]. The participation of calcium metabolism in neurovascular coupling provides a hint for a possible pathologic molecular mechanism that may arise from astrocytic activation. Neuron-to-astrocyte signaling is considered being a key mechanism in functional hyperemia. The resultant increase in extracellular glutamate following astrocytic transformation can activate glutamate receptors (mGluRs) located on astrocytes. It has been shown that the dilation of arterioles triggered by neuronal activity is dependent on glutamate-mediated cytosolic calcium ([Ca^2+^]i) oscillations in astrocytes [127]. Activation of mGluRs and the subsequent elevation in [Ca^2+^]i in astrocytes ultimately creates [Ca^2+^]i increase in the endfeet [128]. Zonta et al. [127] demonstrated that inhibition of astrocytic Ca^2+^responses resulted in the impairment of activity-dependent vasodilation, whereas selective activation of single astrocytes in close proximity to arterioles triggered vessel relaxation [127]. They further observed that in vivo blockade of glutamate-mediated [Ca^2+^]i elevations in astrocytes reduced hyperemic reaction in the somatosensory cortex during contralateral forepaw stimulation. Excess of extracellular glutamate that leads to activation of mGluRs and the increase of [Ca^2+^]i in the endfeet, initiate the activation of Ca^2+^-sensitive K^+^ channels (BK) and the efflux of K^+^. BK channels were proposed to play a role in the K^+^ modulation of cerebral blood flow [129]. Extracellular excess of potassium has the potential to generate changes in the vascular tone through activation of inward rectifying K^+^ channels (K_IR_) located in smooth muscle (SMC) layer of vessel [130]. BK channels, expressed abundantly in astrocytic endfeets, exhibit sensitivity to membrane depolarization and intracellular calcium levels. Neuronal stimulation of brain slices produced BK channel-mediated K^+^ release in astrocytic endfeets, altered the extracellular K^+^([K^+^]o) level in the perivascular space and generated a signal that produces vasodilatory response by K_IR_ channels in parenchymal arteriole SMC. The elevation of [K^+^]o from 3 mmol/L to 8 mmol/L hyperpolarizes parenchymal arteriolar membranes from −45 to −80 mV, and causes a rapid and profound dilation of isolated pressurized parenchymal arterioles as well as arterioles in brain slices [131]. Thus, astrocytic activation after BBB disruption, subsequent reduction in K^+^ buffering and the increase of extracellular glutamate and K^+^, elevates the [K^+^]o levels. This will consequent in enhancement of the mutual activity of glutamate-mediated [Ca^2+^]i oscillations in astrocytes, BK activation and vasodilatation through SMC K^+^ channels. A direct link between the metabolic state in the brain tissue and astrocyte signaling was recently established [132]. According to our hypothesis, the changes in the perivascular microenvironment and the metabolic dysregulation arising from impairment in cerebrovascular response as disturbed or extensive hyperemia may take part in the mechanisms underlying brain pathologies. Hyperemia in the active regions and hypoperfusion of surrounding areas, under some conditions (extent, duration and location) may result in impaired metabolism, inadequate homeostasis preservation, formation of reactive oxygen species and insufficient removal of toxic metabolites. These insults may participate in the evolvement of cognitive or psychiatric illnesses. Mechanisms of abnormal plasticity are also suspected to participate in the development of mental disturbances following BBB breakdown, glial activation and inflammation, via the effect of excess of glutamate. We hypothesize that diffusion of glutamate and K^+^ out of the narrow synaptic cleft will affect neighboring synapses, resulting in a loss of synapse- and pathway-specific plasticity. Astrocyte-mediated plasticity mechanisms utilize glutamate for transient mGluR-dependent neuromodulation. In addition long-term potentiation via NMDAR-independent mechanism showing Ca2^+^ elevation in astrocytes that modulates transmitter release probability and evokes long-term synaptic plasticity [133]. This control in transmitter release at the synapse and the strengthening of synaptic connectivity may possibly result in synaptic tuning in circuits involved in cognitive processing and the control of limbic system excitability [134]. Formation of new synapses may reduce specificity and is expected to activate larger neuronal networks in response to stimuli. These alterations might be expressed in disturbed thinking processes and extreme mood-related behavioral responses, depending on the involved network.

## 10. Conclusion

Inflammation and vascular pathology have a significant contribution for the pathogenesis of T2DM complications. Neuropsychiatric disorders are also associated with inflammatory reaction and cerebrovascular impairments. Brain injuries that often involve BBB breakdown and astrocytic response increase the risk for neuropsychiatric sequelae, including personality changes, depression, anxiety, dementia, and perhaps psychosis [135, 136]. T2DM patients show higher susceptibility to cerebrovascular diseases which according to our hypothesis may explain the increased incidence of cognitive deterioration, depression, vascular dementia, lacunar infarcts, hemorrhages and Alzheimer's disease among these patients. Revealing the mechanisms underlying the effects of diabetes on BBB structure and function and understanding the role of inflammation, impaired neurovascular coupling, metabolic defects and altered neuronal plasticity in the neuropsychiatric sequela of T2DM, will create a target for clinical and pharmacologic modalities and a potential platform for future therapeutic intervention.
